# Anti-VEGF injection frequency correlates with visual acuity outcomes in *pro re nata* neovascular AMD treatment

**DOI:** 10.1038/s41598-019-38934-8

**Published:** 2019-03-01

**Authors:** Thomas Wecker, Bastian Grundel, Sabine Reichl, Milena Stech, Clemens Lange, Hansjürgen Agostini, Daniel Böhringer, Andreas Stahl

**Affiliations:** 1 Eye Center, Medical Center – University of Freiburg, Faculty of Medicine, University of Freiburg, Freiburg, Germany; 2 Ophthalmic practice Dr. Wecker, Heilbronn, Germany; 3 Department of Ophthalmology, University Medical Center, Greifswald, Germany

## Abstract

Clinical trials report substantial gains in visual acuity (VA) for eyes treated with intravitreal anti-VEGF for neovascular AMD (nAMD). In clinical reality, VA outcomes are more variable. Here we investigate pro-re nata treatment frequencies and VA in a real-life cohort of 1382 eyes (1048 patients). Patients with nAMD and one year complete follow-up treated with pro-re nata anti-VEGF between 2009 and 2016 were included. Injection frequency and VA was analyzed clustered by year of first treatment. Baseline parameters were compared between years. Median injection frequency in the first year was 5 with an IQR (interquartile range) of 5 for patients treated in 2009 and 8 with an IQR of 3 for patients treated from 2012 onwards. Median VA outcomes at one year were −5 to ±0 letters for patients treated between 2009 and 2013 and ±0 to +2 letters for patients treated from 2013 onwards. This cohort comprises all severities and subtypes of nAMD. 39% of patients had baseline VA outside the range for the MARINA or ANCHOR clinical trials. Higher treatment frequency was associated with improved VA in our real-life nAMD cohort. With adequate injection frequency, almost 90% of eyes had stable or improved VA over one year. Median VA gains, however, were lower compared to clinical trials. This may be due to a wider range of baseline characteristics in real-life cohorts.

## Introduction

Anti vascular endothelial growth factor (VEGF) treatment has revolutionized the treatment of neovascular age-related macular degeneration (nAMD). Since their introduction into clinical care in 2005, anti-VEGF agents have significantly helped to stabilize and improve vision in hundreds of thousand AMD patients worldwide. However, almost all AMD patients require long-term therapy with frequent intravitreal injections. Several approaches to treat and monitor AMD patients have been developed. They include (i) rigid monthly injections, (ii) pro-re-nata treatments (PRN) with patients only being injected if they show activity on optical coherence tomography (OCT), fluorescein angiography or fundoscopy and (iii) treat-and-extend regimens (TAE) with patients being injected at increasing or decreasing intervals depending on their disease activity. Our center has been following a PRN regimen since 2009 that closely resembles the IVAN study regimen^1^ (see Methods for details). Using this modified IVAN regimen, we have previously published compound results from five years anti-VEGF treatment^2^. The purpose of the current study is to evaluate whether injection frequencies have changed between 2009 and 2017 and whether such changes impact visual acuity (VA) outcomes. In addition, this study describes the differences between a real-life nAMD cohort and clinical trial patients regarding baseline demographics and VA outcomes.

## Methods

**Figure 1:**
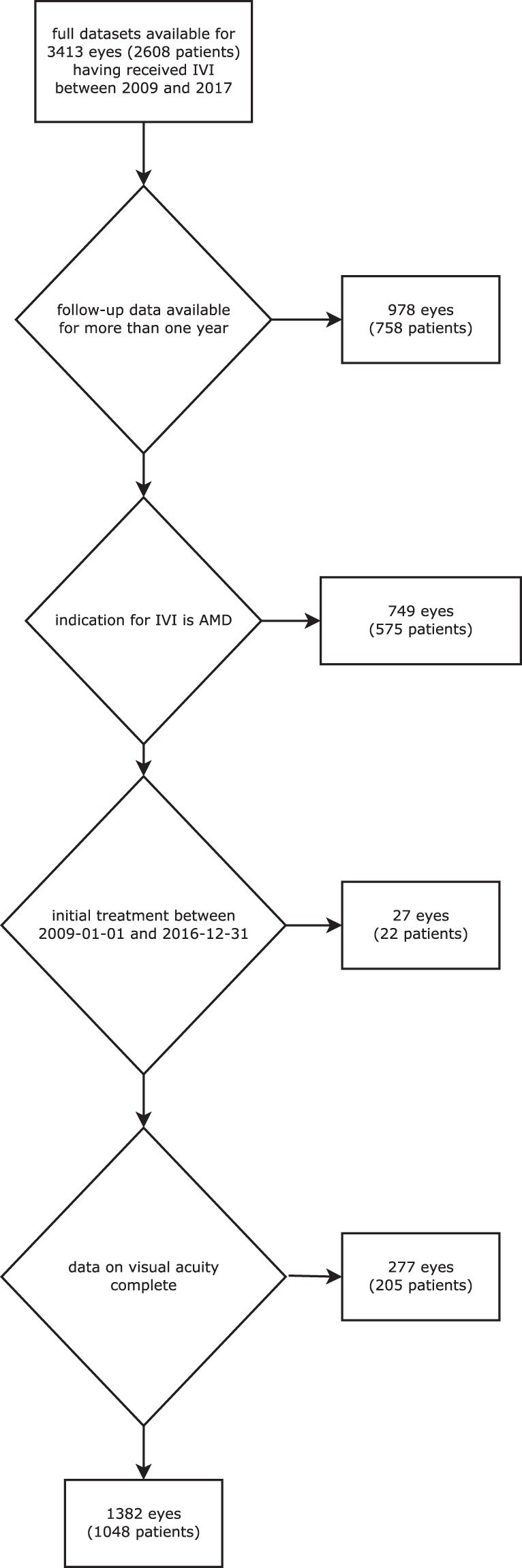
Flow chart of all patients included in the analysis.

Patients being diagnosed with nAMD (represented by International Classification of Diseases (ICD)-code H35.3) receiving their first intravitreal injection between January 2009 and December 2016 were included in this retrospective analysis. The diagnosis nAMD was based on the following OCT findings: drusen in association with subretinal and/or intraretinal fluid +/− pigment epithelial detachment (PED). Active leakage of the CNV membrane was confirmed by fluorescein angiography at first presentation and during follow-up as needed. All patients were treated using a pro-re-nata regimen with sets of three monthly anti-VEGF injections as standard treatment, but adjusted to disease activity based on the examiners judgement (i.e. treatment intervals could be prolonged to 6 weeks if only minimal activity was noted; minimum treatment intervals, however, were always 4 weeks). All decisions to inject as well as on injection frequencies were made based on both OCT and VA findings supplemented by fluorescein angiography where needed. Patients with inactive nAMD were followed for six months with monthly OCT visits. In cases of sustained inactivity beyond 6 months patients were referred back to their primary care Ophthalmologist for further follow-ups. Patients and physicians were free to choose between bevacizumab, aflibercept or ranibizumab and patients could be switched between anti-VEGF substances during treatment. All methods were carried out in accordance with relevant guidelines and regulations. All experimental protocols were approved by the Ethics Board of the University of Freiburg Medical Centre (No. 26/15). This is a retrospective study. All data was anonymized in the first step of data processing. Therefore the need to obtain informed consent was waived by the Ethics Committee. The method of clinical data acquisition has been described previously^2^. Patients with a follow-up period of ≤1 year or without full electronically available visual acuity (VA) measurements were excluded (Fig. 1).

Patients were analysed on a per-eye-basis, i.e. if patients received bilateral injections both eyes were analyzed independently and all analyses reported in this study are per eye. VA was acquired on decimal charts and converted to ETDRS-equivalents for further analysis and comparison. Different from controlled clinical trials this is a real life cohort. This means that a number of different operators collected VA measurements and that operators changed over the years. Autorefraction was performed before each VA exam and VA was recorded with autorefraction values as well as with the patient’s own glasses. The better VA was used for analyses. For VA readings “hand motion” and “counting fingers”, we determined an equivalent as described previously^3^. It should be noted that measured Snellen acuities (or decimal acuities as in our study) may be lower than measured EDTRS acuities in the same eye^4,5^. We have corrected our baseline VA values for comparison with trials using ETDRS charts based on Falkenstein *et al*.^4^ Lines read only partially during decimal VA testing were counted if at least three of five characters were read correctly. Data processing and statistical analysis was done using GNU R and additional packages^6–9^.

### Precis

This study reports anti-VEGF treatment frequencies and visual acuity from a real-life neovascular AMD cohort (1382 eyes). Median injection frequency was 5 to 8.; higher injection frequency was associated with superior visual acuity.

## Results

**Figure 2:**
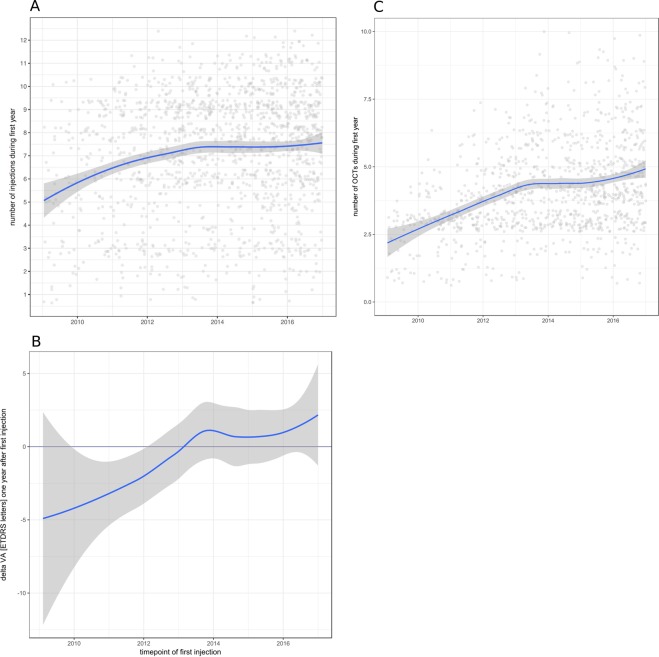
Development of injection frequencies and VA outcomes over time. **(A)** The number of injections in the first year of treatment increased over time. Eyes with their first injection in 2009 received a mean of 5 to 6 injections (median 5). Eyes with treatment initiated after 2013 received a mean of 7.5 injections (median 8) and PRN injection frequencies did not increase further. Graph shows mean injection numbers in blue with 95% confidence interval in grey. Individual eyes are represented by light grey dots. x-axis represents year of first treatment. **(B)** Mean visual acuity at the end of the first treatment year shows a decrease of up to five letters for eyes treated between 2009 and 2013. Eyes treated from 2013 onwards gained on average 0 to 2 letters. Graph shows mean VA acuity change in blue with 95% confidence interval in grey. Individual eyes are represented by light grey dots. x-axis represents year of first treatment. **(C)** Mean number of OCT exams performed in the first treatment year increases over the years mirroring the increase in injection numbers and VA. Graph shows mean number of OCT exams in blue with 95% confidence interval in grey. Individual eyes are represented by light grey dots. x-axis represents year of first treatment. Y-axis is cut at 10 for better visualization; 11 eyes had more than 10 OCTs in the first year (max. 18).

In order to identify possible changes in injection frequencies over time, we separated our data set by time of first injection. Figure 2A demonstrates an increase of median injection numbers over time. In 2009, the median number of injections per eye was 5 with an IQR (interquartile range) of 5. The IQR indicates a high interindividual variability. Between 2010 and 2012, median injection numbers in the first treatment year increased to 8 (IQR 3) and remained on that plateau from 2012 onwards. This increase in mean injection frequency is statistically significant (p < 0.01 for years 2012 and later, Dunnett-test). Interestingly, the increase in injection frequency over the years was mirrored by a similar increase in VA outcomes: patients with treatment initiated in 2009 displayed a median VA loss between −4 and −5 letters at the end of year one while patients treated from 2013 onwards showed a median VA outcome between ±0 and +2 letters (Fig. 2B). It should also be noted that no further increase in injection frequencies was observed from the year 2013 onwards indicating a stable plateau of PRN injections at 8 injections in the first treatment year.

**Figure 3:**
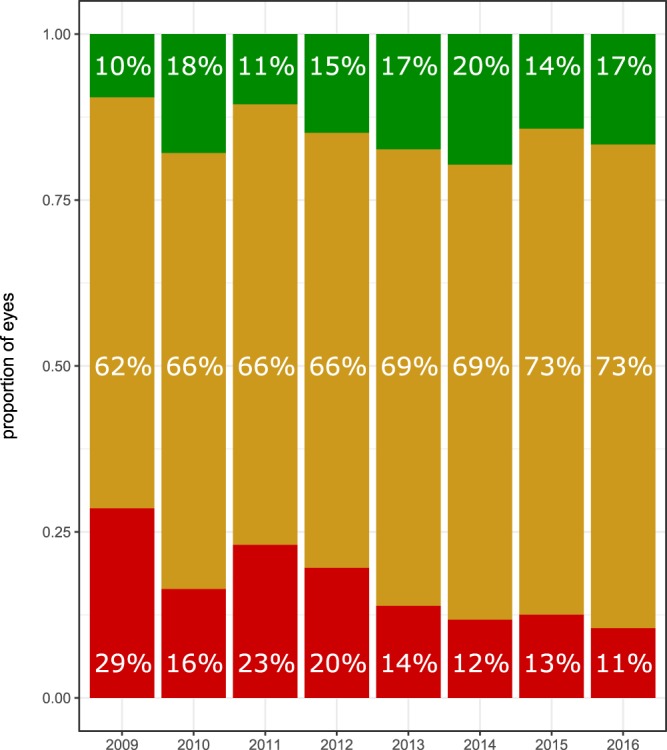
Proportion of eyes with significant VA change. The proportion of eyes with VA gain ≥3 lines (green) was between 10 and 20% in all treatment years with a tendency towards higher proportions from 2013 onwards. The majority of eyes in all years remained within ±3 lines (yellow). The proportion of eyes with VA loss ≥3 lines (red) was between 11 and 29% with a trend towards lower proportions from 2013 onwards.

In all years analyzed, the majority of patients remained within ±3 lines VA change (Fig. 3). The proportion of eyes with significant VA loss (>3 lines), however, decreased from 29% in 2009 to an average of 12.5% between 2013 and 2016 while the proportion of eyes with significant VA gain increased from 10% in 2009 to an average of 17% between 2013 and 2016.

**Figure 4:**
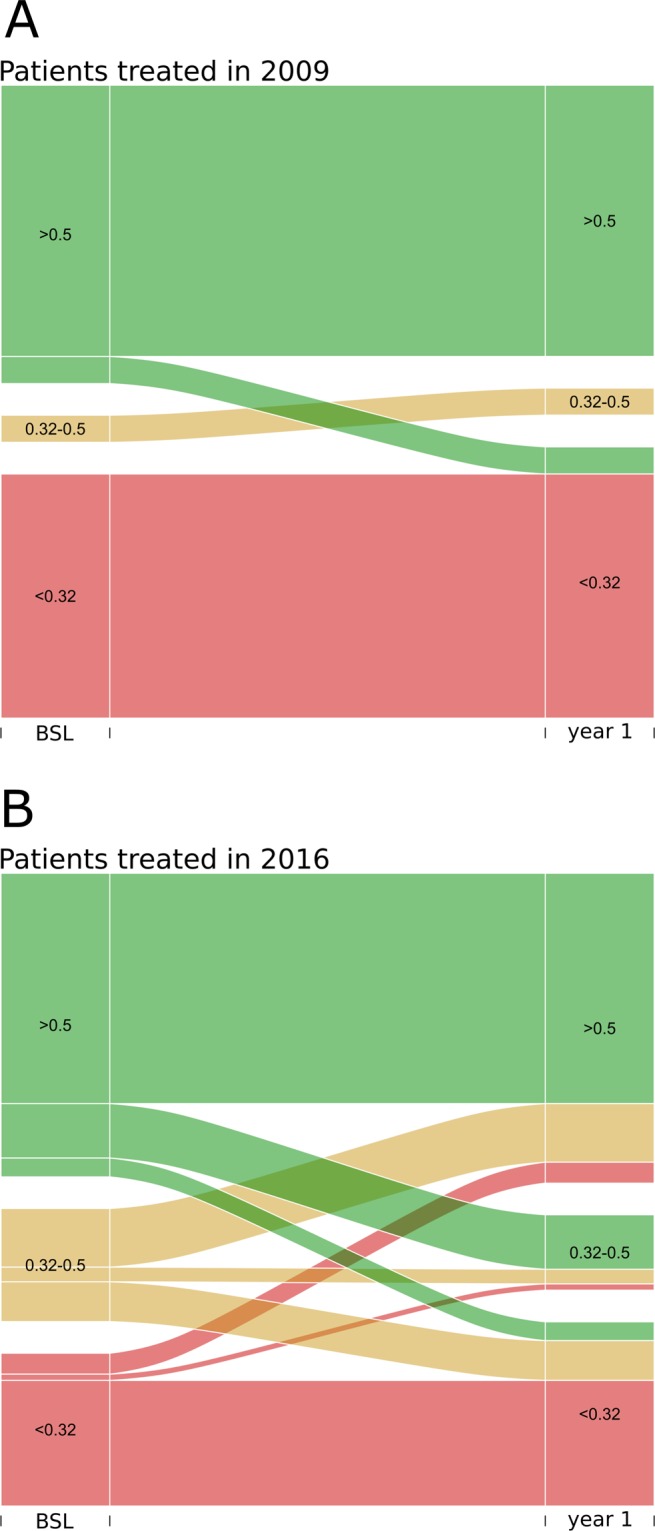
Eyes grouped by VA categories at baseline and end of treatment year. In both exemplary years analyzed, most eyes remained in their baseline treatment categories (green = good VA, yellow = intermediate VA, red = poor VA). In 2009, few eyes from the good VA group deteriorated to poor VA at the end of year one while no eye increased from poor to intermediate or good VA. In 2016, more eyes changed VA groups going from good to intermediate or poor VA and vice versa. Eyes with intermediate VA at baseline improved or deteriorated in comparable proportions while only a minority of eyes remained in the intermediate category.

Achieving certain VA thresholds that are required for performing important everyday tasks like driving a car may be more relevant to patients than absolute VA changes. We therefore analyzed baseline VA and outcomes clustered into three categories: (i) good VA of ≥0.5: able to read and drive; (ii) intermediate VA of 0.3–0.49; and (iii) poor VA ≤ 0.3. Figure 4 displays the proportion of patients in each of the above categories at baseline and at the end of year one. Across all treatment years, most eyes remained in their respective baseline VA category. Comparing the two ends of our analysed time window, however, yields interesting differences. For eyes with treatment initiated in 2009, the only significant group of patients switching categories were eyes moving from VA ≥ 0.5 down to VA < 0.32. For patients with treatment initiated in 2016, the overall proportion of eyes switching VA categories increased and we observed eyes moving up as well as down across VA categories.

**Figure 5:**
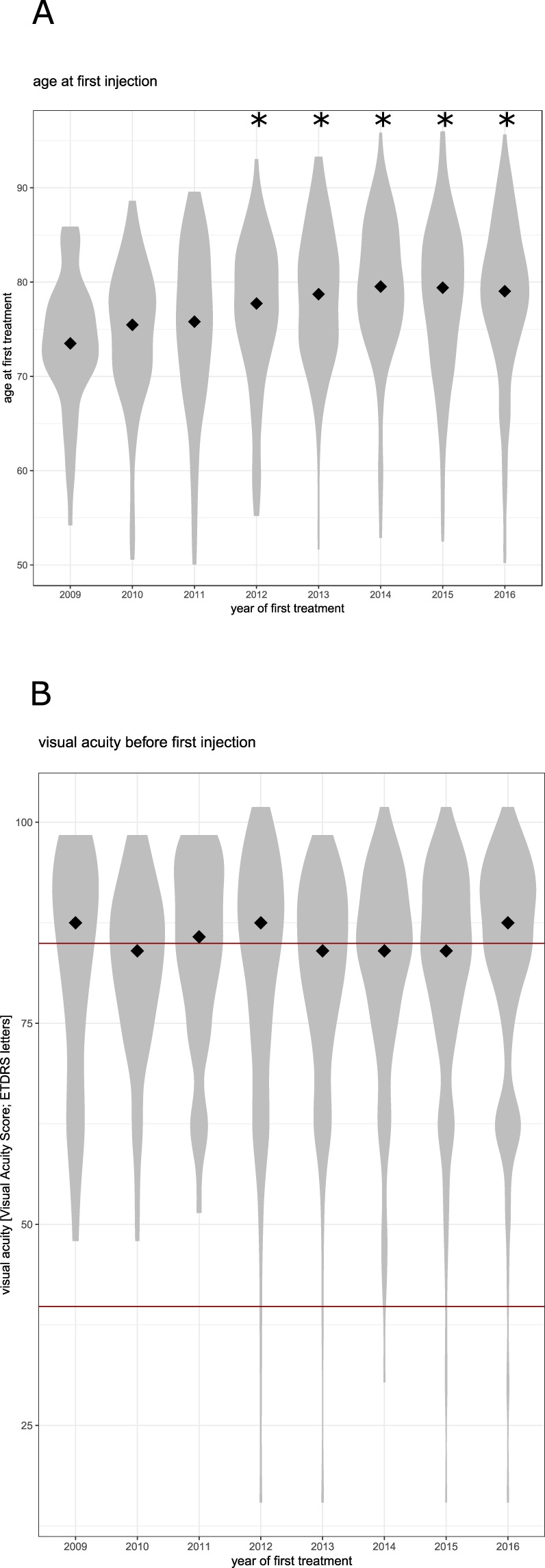
Patient age and VA at baseline. **(A)** Patient age at first treatment slightly increased over the years. Patients from 2012 onwards were statistically significantly older compared to 2009 (*p < 0.05, Dunnet’s t-test). **(B)** Baseline VA did not differ over the years. Horizontal lines show VA inclusion limits for MARINA and ANCHOR clinical trials. Note that only 60.6% of our real life cohort falls into the VA inclusion criteria of these clinical trials. Baseline VA for this analysis was adjusted for differences between decimal and ETDRS measurements based on Falkenstein *et al*.^4^.

We next investigated whether baseline demographic criteria differed between patients over the years (Fig. 5). In 2009, median patient age was 73.5 years (IQR 7.0) and increased slightly in the following years with a peak of 79.52 years (IQR 9.7) in 2014. These differences were statistically significant for all years from 2012 onwards (compared to 2009; Dunnett p < 0.05) (Fig. 5A). Different from patient age, median VA at baseline did not differ significantly between the treatment years (p = 0.44, Kruskal-Wallis-Test; Fig. 5B).

## Discussion

Anti-VEGF treatment significantly improves VA outcome of patients with nAMD^10,11^. The VA gains achieved in clinical trials, however, are often not paralleled in real-life. For example, a recent study of a large US real-life cohort revealed stable mean VA with +/− 0 letters at one year^12^. This is considerably better than the natural history of untreated nAMD but far from the VA gains found in randomized clinical trials (RCTs)^13^. The main difference between real-life data and RCTs is that RCTs represent a highly selected cohort of patients. Besides disease-related inclusion criteria like limited lesion size, fairly good but not excellent VA at baseline and absence of central macular scarring, patients in RCTs must meet other inclusion criteria. For example, RCT patients are selected based on their ability to understand the implications of study participation and their ability to attend frequent study visits. As a consequence, a large proportion of patients requiring treatment in real-life settings are not represented in RCTs. In a real life setting, in contrast, a certain degree of result variability may stem from the heterogeneity of the underlying disease.

In our study, median VA outcome at one year was between −5 and +2 letters from baseline, depending on the year in which anti-VEGF treatment was initiated. Importantly, our treatment paradigm remained the same between 2009 and 2017. We observed, however, that the stringency with which treatment was applied improved over the years. This reflects an improvement in treatment paths and improved patient communications regarding the importance of treatment adherence. Decision to treat was always based on OCT parameters of active nAMD in combination with VA development and backed up by fluorescein angiography as needed. It is known that the required number of injections for nAMD with PRN treatment is around 7 injections per year^14,15^. From 2013 onwards, our data demonstrates a plateau with a mean injection frequency of 7.5 per year. This plateau of adequate anti-VEGF frequency is mirrored by median VA outcomes between 0 and +2 letters from 2013 onwards. Before 2013, our mean injection frequencies were lower (indicating undertreatment) and median VA outcomes ranged between −5 and ±0 letters. Undertreatment is one of the main risk factors for poor VA outcome^16^. Our data confirms these findings. In addition, our data from 2013 onwards provides evidence that with frequent PRN injections, median VA can be maintained in a real-life population that contains all subforms and severities of nAMD. Our data also shows that PRN injections in our cohort did not increase to median values above 8 injections in the first year. It is important to find the right balance between treatment cost, time and benefit for the patient in AMD treatment. Our data indicates that 8 injections in the first year may represent this balance point.

Importantly, a significant proportion of eyes in our real-life cohort had baseline VAs outside the range that would have permittted enrolment into the clinical trials that had led to the approval of anti-VEGF therapies. Only 61% of eyes would have qualified for partizipation in the MARINA or ANCHOR clinical trials. A significant proportion of our eyes showed baseline VA values above the threshold for RCT trial participation. These eyes with higher baseline VA have limited room for VA improvement. In our dataset, 39% of eyes had baseline VA either above or below the standard thresholds for RCTs. These numbers illustrate the importance of real-life studies in anti-VEGF treatment. While for select patients (about 15% in our cohort) substantial VA gains >3 lines can be achieved, a more realistic aim for the majority of patients in a real-life setting is VA stabilization. For patient guidance and treatment adherence, it is important to communicate realistic treatment aims and provide patients with a realistic prognosis regarding VA outcomes. Our data and others show that injection frequency is closely associated with VA outcomes in nAMD^17^ – a fact that should be emphasized during patient communication.

## Data Availability

Compund anonymized data can be made available upon request.
